# Effects of horizontal distance and limb crossing on perceived hand spacing and ownership: Differential sensory processing across hand configurations

**DOI:** 10.1038/s41598-018-35895-2

**Published:** 2018-12-07

**Authors:** Hassan G. Qureshi, Annie A. Butler, Graham K. Kerr, Simon C. Gandevia, Martin E. Héroux

**Affiliations:** 1 Neuroscience Research Australia, Sydney, NSW Australia; 2 University of New South Wales, Sydney, Australia; 3 Movement Neuroscience, Institute of Health & Biomedical Innovation, Queensland University of Technology, Brisbane, Australia

## Abstract

We have previously shown that, with the hands apart vertically, passively grasping an artificial finger induces a sense of ownership over the artificial finger and coming-together of the hands. The present study investigated this *grasp illusion* in the horizontal plane. Thirty healthy participants were tested in two conditions (*grasp* and *no grasp*) with their hands at different distances apart, either crossed or uncrossed. After 3 min, participants reported perceived spacing between index fingers, perceived index finger location, and, for the *grasp* condition, perceived ownership over the artificial finger. On average, there was no ownership at any of the hand configurations. With the hands uncrossed 7.5, 15 or 24 cm apart, there was no difference in perceived spacing between the *grasp* and *no grasp* conditions. With the hands crossed and 15 cm apart, perceived spacing between index fingers was 3.2 cm [0.7 to 5.7] (mean [95% CI]) smaller during the *grasp* condition compared to *no grasp*. Therefore, compared to when the hands are vertically separated, there is an almost complete lack of a g*rasp illusion* in the horizontal plane which indicates the brain may process sensory inputs from the hands differently based on whether the hands are horizontally or vertically apart.

## Introduction

Over the past few decades, important insights about how we sense the position of our body and what body parts belong to us have been obtained by studying disorders and experimental illusions^1,2^. Of particular importance is the rubber hand illusion^2^, where a person’s hand is touched in the same way and at the same time as a visible artificial hand located several centimetres away^2,3^. These two stimuli – touch of the real hand and viewing touch of the artificial hand – are thought to be perceived as a single event^3–6^, which leads to a sense of ownership over the artificial hand and shift in the perceived position of the person’s hand towards the artificial hand^2,7–13^.

We recently showed that with the hands out of view and 12 cm apart in the vertical plane, passively grasping an artificial finger with one hand causes a person to feel their hands are instantly ~5 cm closer^14^. When maintained for 3 min, grasping the artificial finger also induces a general sense of ownership over the artificial finger. In contrast to the rubber hand illusion, our *grasp illusion* occurred without visual feedback or ongoing congruent sensory stimuli. Similarly, the *grasp illusion* differs from the self-touch version of the rubber hand illusion because there was no ongoing congruent sensory stimuli being administered and received by the participant^15^ (see Héroux *et al*.^4^ and Butler *et al*.^16^ for a more detailed comparison of these illusions). Moreover, the person’s hands were spaced vertically. However, many bimanual activities are performed with the hands separated horizontally^12,15,17–19^. While the classic rubber hand illusion can be induced with the real and artificial hand separated vertically, it is not known whether the *grasp illusion*, with its static tactile and proprioceptive sensory signals, is also present when the hands are in a more familiar, horizontal configuration. This is important because a *grasp illusion* in the horizontal plane would highlight the importance of incoming tactile and proprioceptive sensory signals to CNS judgements of body ownership and body representation, independent of upper limb configuration. Conversely, the absence of a *grasp illusion* would indicate the CNS integrates these sensory signals differently when the hands are in a more familiar configuration.

The aim of the present study was to determine whether the grasp illusion occurs when the hands are spaced horizontally. Because distance between the hands influences the strength of the rubber hand illusion^17,20,21^, the impact of distance was investigated. Also, because crossing the limbs increases the strength of the rubber hand illusion^6,18^ and is associated with impaired sensory processing from the hands^22^, the impact of limb crossing was investigated. We hypothesized that passively grasping an artificial finger would induce a sense of ownership over the artificial finger and cause perceived horizontal spacing between the index fingers to decrease. Specifically, we hypothesized that with the hands horizontally 15 cm apart, either crossed or uncrossed, passively grasping an artificial finger would reduce perceived spacing by ~5 cm and induce a sense of ownership comparable to when the hands are vertically apart. These effects were expected to be absent with the hands spaced further apart.

## Results

**Figure 1:**
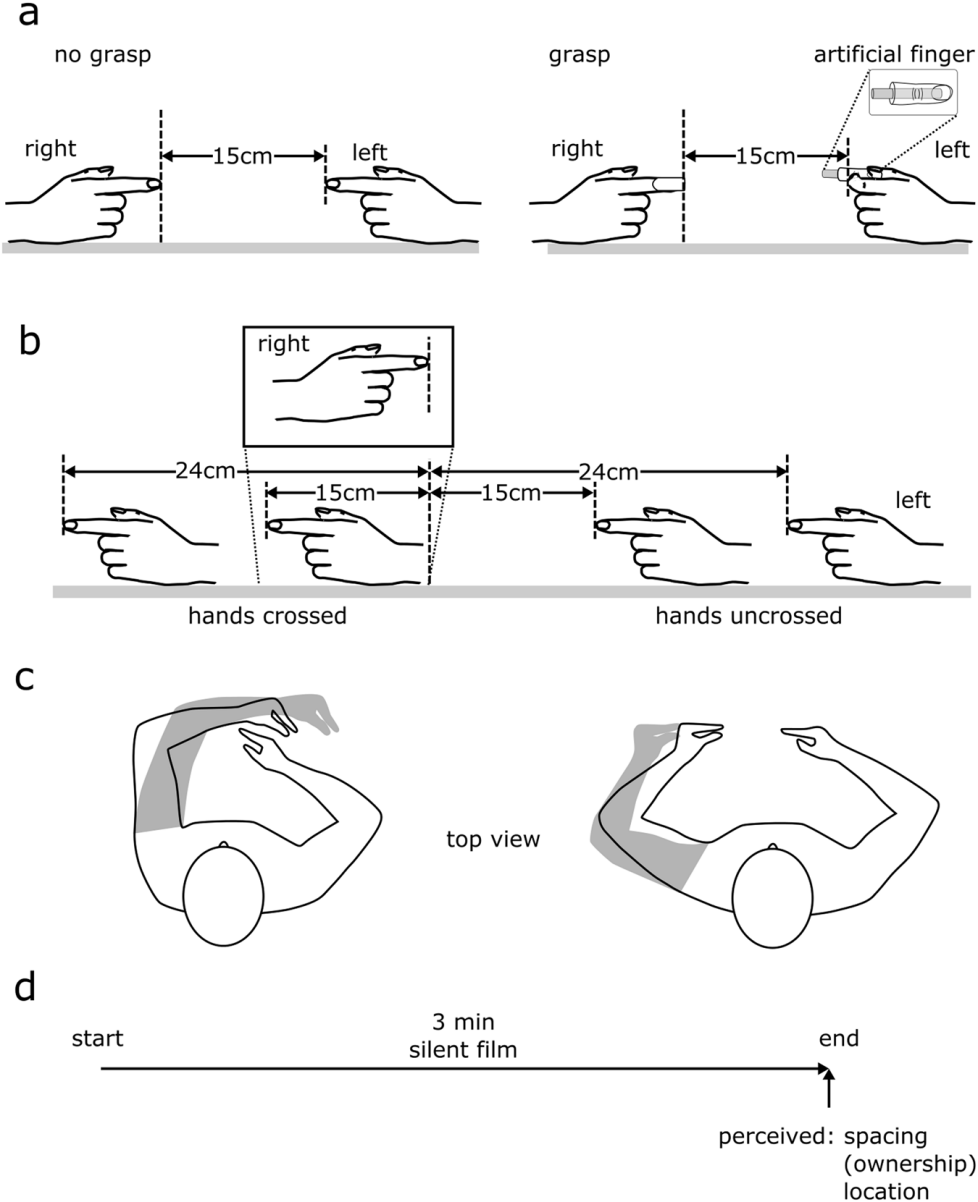
(**a**) Testing took place over two days; each comprised either a *grasp* or *no grasp* condition. For the *grasp* condition, participants passively grasped an artificial rubber finger with their left index finger and thumb, while their right index finger was passively grasped by a clamp. In the *no grasp* condition, the hands were placed in the same positions but without a grasp. The index fingers were at the same height. For clarity, moulds used to support the hands are not shown. (**b**) The *grasp* and *no grasp* conditions were tested with the hands placed in 4 different configurations. The right index finger always remained at the body midline, while the left index finger was at one of the four positions: 15 cm and 24 cm uncrossed, 15 cm and 24 cm crossed. (**c**) Top view of 4 different configurations. (**d**) Each trial lasted 3 minutes during which participants watched a silent film. At the end of the film clip, perceived horizontal spacing between the index fingers, perceived ownership (*grasp* condition only), and perceived location of left and right index fingers were measured (see Methods).

In 29 healthy individuals we investigated the effect of passively grasping an artificial finger for 3 min on perceived horizontal spacing of the index fingers and perceived ownership over the artificial finger. The participant’s right index finger was located at the body midline for each trial. The participant’s left hand was positioned such that the distance between the tips of the left and right index fingers was 15 cm or 24 cm, and this with the hands crossed or uncrossed (Fig. 1).

**Figure 2:**
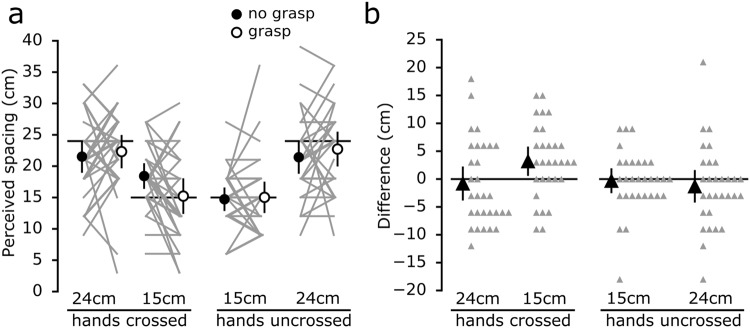
(**a**) Perceived spacing between the tips of the index fingers during *grasp* and *no grasp* conditions for the four horizontal positions of the left hand. The circles depict the group mean for each position, while the error bars show the 95% CI. The solid grey lines connect each participant’s responses for the two conditions. The solid horizontal lines represent actual spacing between the index fingers. (**b**) Differences in perceived spacing between the tips of the index fingers (*no grasp - grasp*) for the four horizontal positions (i.e. a positive value indicates that the fingers were perceived closer together during the *grasp* condition). With the left hand placed 15 cm across the body midline, participants on average perceived their hands to be 3.2 cm [0.7 to 5.7] closer during the *grasp* condition. The big triangles depict the group mean differences in perceived spacing between the two conditions for each position, while the error bars represent the 95% CI. The small triangles show individual differences in perceived spacing between the two conditions.

**Figure 3:**
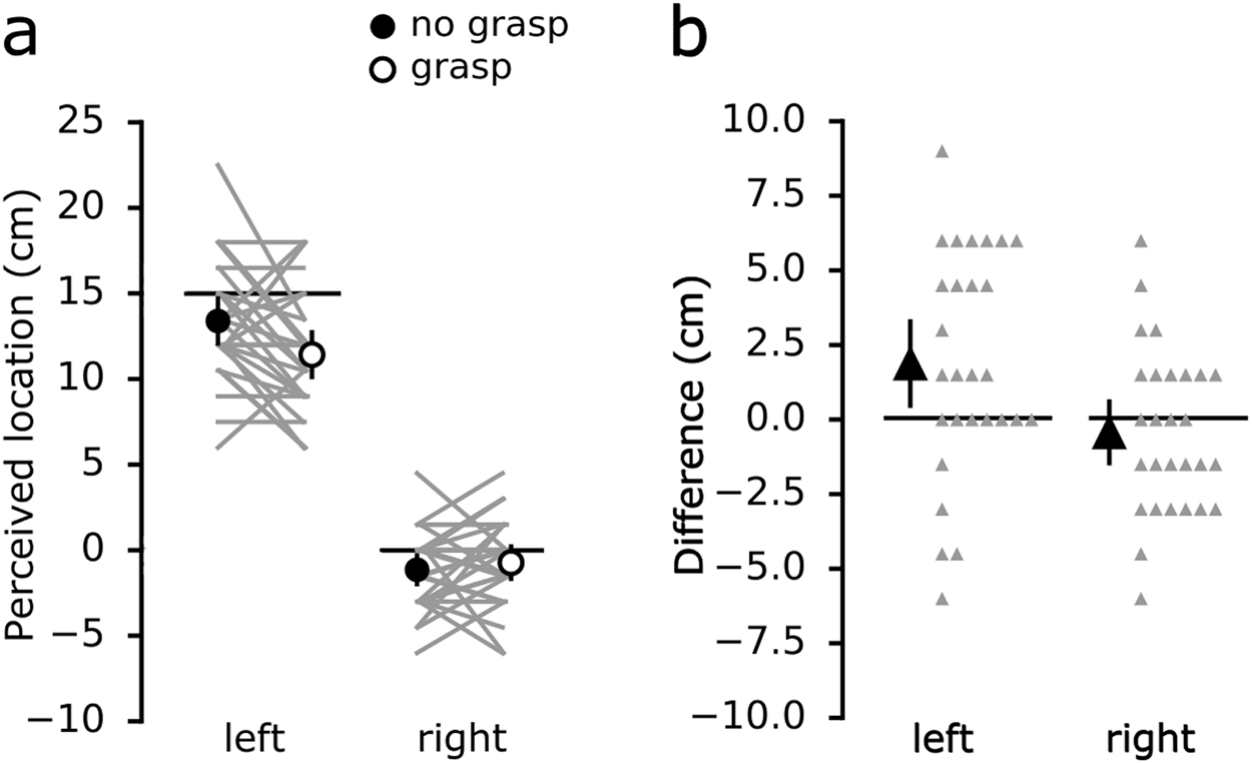
(**a**) Perceived location of the tip of left and right index fingers during *grasp* and *no grasp* conditions with the hands 15 cm apart and crossed. The circles depict the group mean for each condition, while the error bars represent 95% CI. The grey lines connect each participant’s responses for the two conditions. The solid horizontal lines represent actual position of index fingers. (**b**) Differences in perceived location (*no grasp - grasp*) for each hand with the hands 15 cm apart and crossed. Participants on average perceived their left hand to be 1.9 cm [0.5 to 3.3] towards the right hand, while the right hand location was not different between the two conditions (−0.4 cm [−1.5 to 0.6]). The big triangles show the group mean, while the error bars represent 95% CI. Smaller triangles represent individual differences.

**Figure 4:**
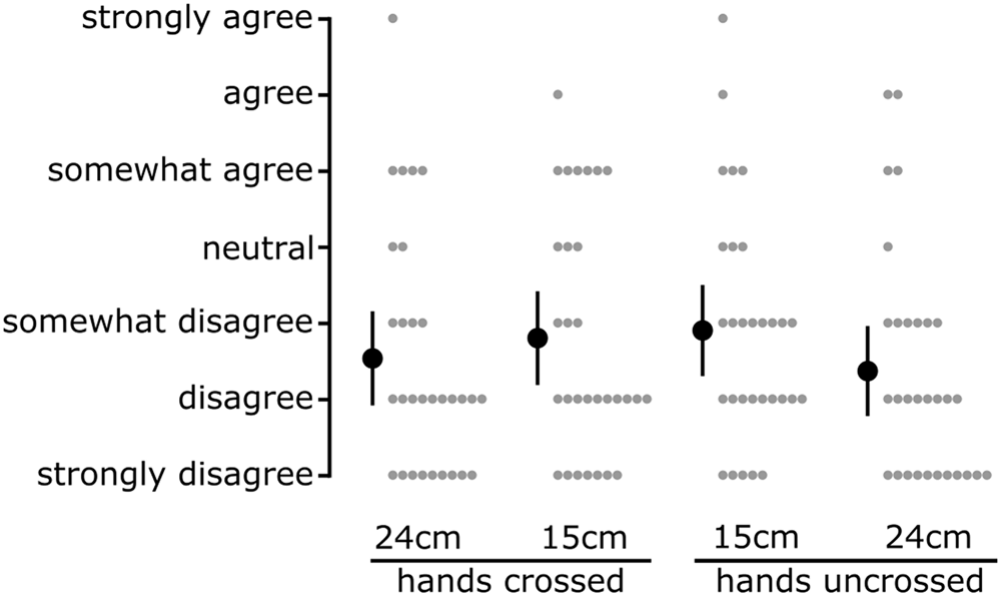
Perceived body ownership over the artificial finger for the four horizontal positions on a 7-point Likert scale. The big circles depict the group mean for each position, while the error bars represent 95% CI. The small circles show individual ratings of perceived ownership.

On average, perceived horizontal spacing was greater when the hands were 24 cm apart compared to 15 cm apart, both when crossed and uncrossed (Fig. 2a). For each of the four hand configurations, differences in perceived index finger spacing between the *grasp* and *no grasp* conditions were determined (Fig. 2b). With the hands crossed 15 cm, passively grasping an artificial finger reduced perceived horizontal spacing by 3.2 cm [0.7 to 5.7] (mean [95% CI]). This effect was associated with the left index finger being perceived 1.9 cm [0.5 to 3.3] more to the left (i.e. towards body midline and the right index finger) during the *grasp* condition compared to the *no grasp* condition (Fig. 3a,b); perceived location of the right index finger was not different (−0.4 cm [−1.5 to 0.6]). Bayes factors for these results are reported in Table 1. Across all four hand configurations, perceived ownership of the grasped artificial finger was, on average, between ‘disagree’ to ‘somewhat disagree’ (Fig. 4).

As part of our pre-planned analyses, we determined the effect of hand spacing amplitude (15 cm *vs* 24 cm) when the hands were crossed and uncrossed. With the hands crossed, differences in perceived index finger spacing between the *grasp* and *no grasp* conditions were 3.9 cm [0.7 to 7.2] greater when the hands were spaced 15 cm compared to 24 cm. No such effect was present when the hands were uncrossed (0.8 cm [−2.0 to 3.7]). Perceived ownership was not affected by hand spacing when the hands were crossed (0.2 [−0.2 to 0.7]), however there was a small effect with the hands uncrossed (0.5 [0.2 to 0.8]). We also determined the effect of hand configuration (crossed *vs* uncrossed) when the hands were 15 cm and 24 cm apart. With the hands spaced 15 cm, differences in perceived index finger spacing between the *grasp* and *no grasp* conditions were 3.5 cm [0.8 to 6.2] greater with the hands crossed compared to uncrossed. No such effect was present when the hands were spaced 24 cm (0.4 cm [−2.4 to 3.2]). Perceived ownership was not affected by hand configuration whether the hands were 15 cm (−0.2 [−0.7 to 0.2]) or 24 cm (0.1 [−0.4 to 0.5]) apart. Although hand spacing amplitude and hand configuration influenced differences in perceived index finger spacing between the *grasp and no grasp* conditions, these results are dominated by the effect with the hands crossed and 15 cm apart (Fig. 2b).

**Figure 5:**
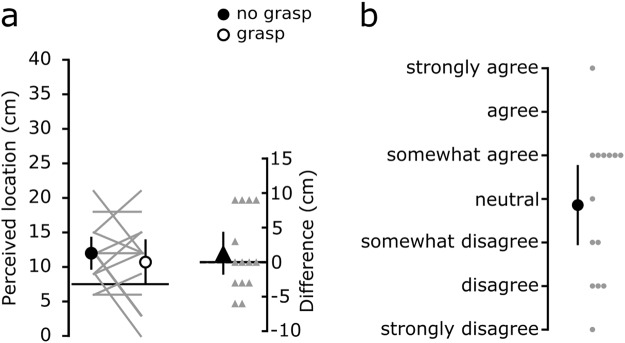
(**a**) Perceived spacing between the tips of the index fingers during *grasp and no grasp* conditions with the hands 7.5 cm apart and uncrossed. The circles depict the group mean for each condition, while the error bars show the 95% CI. The grey lines connect each participant’s responses for the two conditions. Secondary axis depicts difference in perceived spacing between the tips of the index fingers (*no grasp* - *grasp*) with the hand 7.5 cm apart and uncrossed. The big triangle depicts the group mean difference in perceived spacing between the two conditions, while the error bars represent the 95% CI. The small triangles represent individual differences in perceived spacing between the two conditions. (**b**) Perceived body ownership over the artificial rubber finger with the hands 7.5 cm apart and uncrossed on a 7-point Likert scale. The big circle depicts the group mean, while the error bars represent 95% CI. The small circles represent individual ratings of perceived ownership.

Given our previous results of a *grasp illusion* with the hands spaced 12 cm apart in the vertical plane^14^, we were surprised at the lack of effect when the hands were uncrossed and 15 cm apart. Therefore, we conducted a follow-up experiment to determine whether grasping an artificial finger for 3 min with a smaller horizontal spacing (7.5 cm, uncrossed) would lead to a sense of ownership over the artificial finger and reduced perceived index finger spacing. In 14 healthy individuals who participated in the first part of the study, the difference in perceived index finger spacing between the *grasp* and *no grasp* conditions was 1.3 cm [−1.8 to 4.4]) (Fig. 5a). The 95% CI is large and includes 0 cm, indicating the lack of a systematic effect across participants. Bayes factor for this result is reported in Table 1. Similarly, perceived ownership was, on average, between ‘somewhat disagree’ to ‘neutral’.

## Discussion

We investigated whether passively grasping an artificial finger with the hands horizontally apart reduces perceived spacing between index fingers and induces a sense of ownership over the artificial finger. Surprisingly, participants did not experience a sense of ownership over the artificial finger in any of the tested hand configurations, even in the follow-up experiment where the hands were uncrossed and the tips of the index fingers were only 7.5 cm apart. Only at one of the five hand configurations, passively grasping the artificial finger caused a small (i.e. ~3 cm) decrease in perceived index finger spacing.

Why was there no clear *grasp illusion* with the hands spaced horizontally when our previous work found a clear illusion with the hands spaced vertically? Brain processes involved in the sense of body ownership are thought to be driven by Bayesian sensory inference^3,5,16^. In this model, temporal and spatial congruence of sensory signals, integrated in the context of prior experiences, influence the likelihood that these sensory signals are perceived to have a common cause. Thus, the lack of a *grasp illusion* with the hands spaced horizontally could be due to a difference in incoming sensory signals (cutaneous, proprioceptive) or a difference in the prior probability that these sensory signals have a common cause (‘I am holding my own index finger’).

Because the *grasp illusion* involves primarily static sensory signals, spatial congruence is key. During the experiment, cutaneous inputs signalled that the right index finger was being grasped and that the left index finger and thumb were grasping a finger-like object. The artificial finger and the clamp holding the subject’s right index finger were designed to provide cutaneous feedback similar to real human fingers, and they were applied to congruent locations on either hand. Thus, cutaneous inputs had high spatial congruence. Conversely, proprioceptive signals indicated the position and configuration of both upper extremities, information used by the CNS to localise cutaneous inputs in three-dimensional space by remapping skin-based coordinates to external space as a function of body posture^23^. The extent to which the right and left hands were perceived to be next to one another will have influenced the likelihood the CNS assigned a single cause to the tactile signals arising from the right and left hands. That the hands were perceived juxtaposed to one another was favoured by the tendency to erroneously perceive the hands closer to midline^5,11,24^. This effect may have been further strengthened by having the right index finger always located at body midline, possibly acting as an anchor or positional reference to the middle of the body; an anchor that is typically not present in the rubber hand illusion, which may favour the presence of an illusion. Despite this and despite the fact we included a condition in which the index fingers were only 7.5 cm apart, no *grasp illusion* was observed. Why? It could be that, compared to when the hands are spaced vertically, people are more accurate at locating horizontally spaced hands. This greater accuracy would signal the CNS that the hands are apart and decrease the likelihood cutaneous signals from the right and left hands (i.e. touching and being touched) have a single cause (i.e. “I am holding my own index finger”). Although plausible, there is currently no evidence to support (or refute) such a vertical-horizontal difference in proprioceptive acuity.

An alternative explanation based on Bayesian sensory inference for the lack of a *grasp illusion* is that the prior probability of assigning a single cause to these sensory signals was lower for horizontally spaced hands. Why would that be? The majority of daily tasks require some level of bimanual interaction, much of which occurs with the hands side-by-side. Whether slicing onions or unclasping a necklace, our hands are often next to each other without necessarily touching, or having one hand grasp the other hand’s index finger. Thus, given the multitude of things we do with our hands in this configuration that do not involve grasping our own index finger, the prior probability associated with ‘I am holding my own index finger’ was likely low. In contrast, it is less common to use our hands one above the other. Thus, although still an unlikely event, the prior probability associated with ‘I am holding my own index finger’ was likely higher for this hand configuration used in our previous study. At present, this explanation is hypothetical. Future work should determine whether the hands and their relative side-to-side and up-down position influence how incoming sensory stimuli are processed and integrated. Relevant to this future work are the recent results of Romano *et al*.^25^ who demonstrated that humans likely have a central brain model of the default spatial configuration of the body that guides perception. Proprioceptive and visual information about the current configuration of our body is automatically integrated in the context of this preferential spatial representation of the body, with experimental evidence that this internal default representation is more heavily weighted for perceptions when visual cues are unavailable. Thus, it might be that the brain’s default spatial configuration for the two hands is uncrossed, possibly even at the same vertical level.

Despite the general lack of a *grasp illusion* in the present study, perceived index finger spacing did decrease 3–4 cm in the *grasp* condition compared to *no grasp* when the hands were crossed and 15 cm apart. The size of this effect is comparable to similar illusions that induce changes in perceived limb position^2,12,14,26,27^. Moreover, a version of the rubber hand illusion involving both hands without visual feedback induces 2–3 times greater changes in perceived hand position when the hands are crossed^6,18^. Why would crossing the hands induce a stronger illusion? Why would crossing the hands be the only condition where we observed an effect of grasping an artificial finger? As highlighted by Azañón *et al*.^28^, our bodies are symmetrical along the right-left axis, with every body location having a contralateral homologue^28^. This characteristic is thought to make it more difficult to determine the left-right position of cutaneous stimuli. Importantly, locating stimuli is more difficult when the hands are crossed due to the conflict between skin-based coordinates of each hand and the coordinate system used to map external space^22^ (for a review see Heed *et al*.^29^). The added uncertainty regarding the location of cutaneous stimuli is thought to increase reliance on the tactile stimuli themselves, which in our study were spatially congruent, and in the above mentioned version of the rubber hand illusion were also temporally congruent^6^, both of which would favour the presence of an illusion (i.e. cutaneous signals from both hands having a common cause). It is not clear why this decrease in perceived index spacing was not accompanied by a sense of ownership. However, a change in perceived limb position with no accompanying ownership is not uncommon with the rubber hand illusion^11,12,30^. Why was there no effect when the hands were crossed 24 cm? The rubber hand illusion decreases in intensity and eventually disappears as the distance between the real and artificial arm increases^5,17,20^; a similar process was likely at play in the present experiment.

The rubber hand illusion shows that ongoing dynamic stimuli need to be synchronised in time and space if they are to alter what we believe is part of our own body. The *grasp illusion* provides evidence that these dynamic stimuli are not required to alter our sense of body ownership and body representation. Here, using the *grasp illusion*, we provide evidence that these bodily senses and the central processes that govern them may be sensitive to the whether the hands are positioned in the vertical or horizontal plane, which may be of some importance when treating individuals with disorders of body ownership.

## Methods

Thirty healthy adults participated in the main part of the study, 15 of whom participated in a follow-up experiment. Participants from the main part of the study were selected pseudo-randomly until the predetermined number of participants were recruited. On average, testing for the follow-up experiment took place 4 months after the main part of the experiment. The data from one subject were later excluded from both studies because abnormal neurological signs were noted during an unrelated neurophysiological study. Thus, 29 healthy adults participated in the main part of the study (13 males, mean age 31.7 years, range 21–57, 3 left handers), 14 of whom participated in the second part of the study (8 males, mean age 29.5 years, range 21–46, 2 left handers). Participants were informed about the general experimental procedure but remained naive to the study’s hypotheses. Participants were offered $20 reimbursement for their participation. All participants provided written informed consent prior to participating. The studies were conducted in accordance with the Declaration of Helsinki (2008) and approved by the University of New South Wales Human Research Ethics Advisory Panel.

### Experimental Set-up

Participants were seated at a table inside an enclosed booth. A cloth ensured participants could not see their hands or arms throughout the study. A monitor (height: 60 cm, width: 105 cm) was located ~70 cm in front of participants, with the base of the screen at approximately shoulder height. The study was conducted using custom software written in the Python programming language.

Participants started each trial with their hands by their sides. Next, the experimenter guided the participants’ forearms and hands to a table at chest height. The hands were placed in low-profile support moulds to ensure correct hand posture was maintained throughout each trial. The hands were in a semi-pronated posture with the index fingers extended and pointing towards the contralateral side (i.e. left index pointed right, right index pointed left) (see Fig. 1a). Both hands were positioned on the same horizontal surface and the tips of the fingers were aligned in the anterior-posterior plane. The tip of the right index was always at the body midline (i.e. frontal plane), while the location of the tip of the left index finger was varied each trial.

In the *no grasp* condition, participants’ hands were positioned as described above. In the *grasp* condition, the tip of the participants’ right index finger was lightly clamped (see Fig. 1b). The experimenter positioned the participants’ left thumb and index finger on an artificial finger and then lightly clamped them to ensure the participants maintained a passive grasp. Both clamps were lined with silicone and participants verbally instructed the experimenter to adjust the clamps to achieve a comfortable, equal pressure on both hands. Participants were instructed to keep their hands and arms relaxed throughout.

### Experimental Measures

The *grasp illusion* was quantified by measuring perceived horizontal spacing between the participants’ left and right index fingers and perceived ownership over the artificial finger. To investigate the change in perceived location of each hand separately, we also measured the perceived location of the tip of each index finger. All measures were taken at the end of the 3 min video (Fig. 1d).

#### Perceived horizontal spacing

The experimenter read out the following question as it appeared on the monitor: ‘Which line corresponds to the horizontal distance between the tips of your index fingers?’. Next, 22 horizontal lines of varying lengths, each labelled with a different letter, were presented on the monitor. The horizontal lines were centered on the monitor and ranged from 0–31.5 cm in length in 1.5 cm increments. The lines were presented in a different order for each trial. Once the participants selected a line, they were asked whether their hands were “crossed or uncrossed”.

#### Perceived body ownership

For the *grasp* condition, the experimenter read out the following statement as it appeared on the monitor: “I feel like I am holding my right index finger with my left hand”. A 7-point Likert scale (‘strongly disagree’, ‘disagree’, ‘somewhat disagree’, ‘neutral’, ‘somewhat agree’, ‘agree’, ‘strongly agree’) appeared on screen and the experimenter asked participants to rate how strongly they agreed or disagreed with the statement. The items from the Likert scale were paired to a linear integer scale (‘strongly disagree’ = 1, ‘strongly agree’ = 7) for analysis.

#### Perceived index finger location

A ruler was placed in front of the participants at shoulder height, 15 cm directly above their hands. The ruler had 49 vertical lines at intervals of 1.5 cm, each labelled with a number. The experimenter read out the following question as it appeared on the monitor: ‘What is the horizontal location of the tip of your left index finger?’ Participants were asked to choose the line which they felt corresponded to the horizontal location of the tip of their left index finger. They were then asked to report the location of the right index finger. Rulers with different numbers were presented for each trial.

### Experimental Protocol

Testing consisted of two conditions, *grasp* and *no grasp*. Each condition was tested on a separate day, one week apart. For the main part of the study, four hand configurations were tested for each condition. With the tip of the right index finger always at body midline, the tip of the left index was either 15 cm or 24 cm to the left (uncrossed) or right (crossed) of the right index finger (Fig. 1b). Condition and trial order were randomised across participants.

Each trial started by positioning participants’ hands. Next, participants watched a 3 min clip from a silent movie, which was immediately followed by measures of perceived index finger spacing, perceived ownership (*grasp* trials only) and perceived index finger location. Following the measures, the experimenter guided the participants’ hands away from the table. Participants placed their hands by their side until the following trial, with a 90–120 s break between trials. The silent clips were used to standardise attention between participants and across trials. There was continuity in the video clips of subsequent trials to encourage participants to attend to them.

Results from the main part of the study indicated there was no *grasp illusion* with the hands uncrossed and spaced 15 cm. This finding could reflect that tactile and proprioceptive sensory signals that led to the *grasp illusion* in our previous study are processed differently when then hands are in a more familiar side-by-side configuration. However, in our previous study the hands were spaced 12 cm; the additional 3 cm used in the present study were required to allow for the crossed condition. The increased distance between the index fingers could have contributed to the lack of a *grasp illusion*. To address this possibility, we conducted a follow-up experiment where the hands were uncrossed and the tips of the index fingers were 7.5 cm apart. Only measures of perceived index finger spacing and perceived ownership were taken for the follow-up experiment. As before, *grasp* and *no grasp* conditions were tested on two experimental days, one week apart.

### Data Analysis

All analyses were pre-planned and no additional analyses were carried out. Data were analysed using an estimation approach based on confidence intervals^31,32^. All values reported in the text and figures are mean [95% CI]. We have not used null-hypothesis significance testing, however a 95% CI for a difference score that does not include zero can be considered statistically significant (p < 0.05)^31^. All analyses were performed with the Python programming language.

We estimated the mean difference [95% CI] in perceived horizontal spacing between *grasp* and *no grasp* conditions for each hand configuration. We also estimated the mean value [95% CI] of perceived ownership for the *grasp* condition of each hand configuration. These represent the two primary outcomes.

If any of the mean differences [95% CI] in perceived spacing did not include zero, indicating that grasping the artificial finger influenced perceived spacing, we estimated the mean difference [95% CI] in perceived index finger location for the right and left index fingers. This allowed us to determine whether the change in perceived spacing was associated with a change in the perceived position of the left index finger, the right index finger, or both index fingers. Positive values correspond to the index finger being mislocalized towards the right when grasping the artificial finger.

The effect of hand spacing amplitude on the primary outcomes was assessed independently for the crossed and uncrossed conditions. We compared perceived spacing difference values (i.e. *grasp* – *no grasp*) between the 15 cm and 24 cm conditions when the hands were crossed, and when they were uncrossed. A similar comparison was performed for perceived ownership values.

The effect of hand crossing on the primary outcomes was assessed independently for the 15 cm and 24 cm hand configurations. We compared perceived spacing difference values between the crossed and uncrossed conditions when the hands were spaced 15 cm apart, and when they were spaced 24 cm apart. A similar comparison was performed for perceived ownership values.

To better interpret our results, especially those where little to no effect was present, Bayes factors were computed for all reported *grasp* – *no grasp* comparisons of perceived spacing and perceived location (Table 1). This was suggested by an anonymous reviewer, thus it was done *post-hoc*. The Bayes factor measures the strength of evidence for the null model (i.e. there is no difference between *grasp* – *no grasp*) relative to the alternative model (i.e. there is a difference between *grasp* – *no grasp*)^33,34^. For all computed Bayes factors, the normally distributed alternative model was based on the pooled effect size estimate from our previous study^14^ and a recently completed study involving another 30 participants (mean = 5.3 cm, standard deviation = 5.3 cm). A Bayes factor of 5, for example, indicates the data are five times more likely given the alternative model; conversely, a Bayes factor of 0.2 (1/5) indicates the data are five times more likely given the null hypothesis^33^. As recommended by Lakens *et al*.^33^, we also report robustness regions that correspond to the lower and upper effect size for Bayes factors of 1/3 and 3. Effect sizes smaller than the lower limit support the null hypothesis, those larger than the upper limit support the alternative hypothesis. Effect sizes that fall within the robustness region are considered inconclusive. Bayes factors were computed with the free online calculator associated with Dienes^34^ and standard errors were corrected for degrees of freedom <30^34^.

**Table 1:** Bayes Factor for perceived spacing and location outcomes.

Condition	Mean	SEM^*^	B_N(5.3,5.3)_^†^	Robustness Regions
Crossed 24 cm spacing	−0.62	1.42	0.16	1.40 to 3.19
Crossed 15 cm spacing	3.52	1.23	12.86	1.40 to 2.85
Uncrossed 24 cm spacing	−1.09	1.34	0.17	1.40 to 3.05
Uncrossed 15 cm spacing	−0.62	1.02	0.12	1.32 to 2.46
Uncrossed 7.5 cm spacing	1.29	1.44	0.30	1.40 to 3.23
Crossed 15 cm Left index location	1.91	0.68	5.38	1.08 to 1.77
Crossed 15 cm Right index location	0.62	0.53	0.13	0.93 to 1.44

*Corrected standard error of the mean (SEM).

^**†**^Bayes factor.

## Data Availability

All data and computer code used for this study is available from github.com/MartinHeroux/SciReports_HorizGraspIllusion2018.
